# Mr. Forbes, on the Distemper of Dogs

**Published:** 1801-10

**Authors:** William Forbes

**Affiliations:** Of the Royal College of Surgeons


### To Dr. BRADLEY.

Sir,

If the incloſed letter to Dr. Jenner meets your views, the inſerting it will much oblige, Sir,

Your obedient ſervant,
WILLIAM FORBES,
Of the Royal College of Surgeons.

---

### To Dr. JENNER.

Sir,

I take the liberty, through the medium of the Phyſical Journal, of informing you that I have ſatisfied myſelf with the Cow-pox being a complete preventive of the Small-pox, if the Vaccine fluid be taken at an early period, and afterwards found to bear the true character, in the patient to whom it was communicated. I have expoſed many ſo treated to the infection of the Small-pox by inoculation; and although the variolous inflammation perfectly ſucceeded, and, I have no doubt, would have infected others inoculated with the fluid ſecreted, yet the conſtitutional progreſs was as completely arreſted as if the patient had gone through the Small-pox before.

After the moſt minute enquiry, no authentic inſtance has as yet occurred, of any having previouſly undergone the Vaccine Inoculation, becoming afterwards the ſubject of the infection of the Small-pox; about this I am the more particular, as many caſes of chicken-pox have been miſtaken for it.

Having lately extended my obſervations to the efficacy of the Cow-pox in preventing the diſtemper incident to dogs, laſt May I inſerted the Vaccine fluid three times into a young female ſpaniel of my own; twice in the inſide of each ear, and once in the inſide of each thigh, but without producing any effect; and the animal now labours under the diſtemper. At the ſame time I inoculated a young female pug, and a young male male ſpaniel, in the inſide of each thigh; theſe were completely vaccinated, but, unfortunately, both of them have ſince had the diſtemper; and while the ſpaniel is now ſlowly recovering, the pug has fallen a victim to its influence. Great attention was paid to this little creature by the lady to whom it belonged; ſhe obſerved the part inoculated to go through every ſtage which is deſcribed as neceſſary for giving it proper effect, the animal never appearing in the ſlighteſt degree ill.

I had intended to have carried my experiments on a more extended ſcale; but theſe two caſes appearing ſo concluſive, and your expreſſing a deſire to Mr. Creſpigny to be acquainted with the reſult, will, I hope, be accepted as an apology for laying them before you.

I ſhall take the liberty to ſubjoin the appearances on opening the animal. The parts exhibiting marks of diſeaſe, were the membrane lining the noſe, the larynx and trachæa, which were in an inflamed ſtate; it is worthy of remark, that the left ſide only of that tube through its whole length was very vaſcular, while the membrane on the right ſide did not deviate from the natural ſtate. The brain was, to appearance, ſound. The inflammation of the mucus membrane, in my opinion, readily accounts for the primary ſymptoms, as affection of the eyes, running at the noſe, ſneezing, and that peculiar kind of cough which ſo particularly characterizes this diſeaſe;* and effects becoming probable cauſes from the diſtreſs and debility induced, vomiting, purging, fits, ſpaſmodic twitching, and apparent paralytic affection, emaciation, &c. ſupervene.

I remain, Sir,

With the greateſt reſpect, &c.

WILLIAM FORBES.

*Camberwell,*

*Sept* 10, 1801.

---

### *To the Editors of the Medical and Phyſical Journal.*

Gentlemen,

THE following relation of a caſe of ſuſpended animation which fell under my own immediate cognizance, being ſomewhat remarkable for the ſubject of it recovering ſufficiently to expreſs his ideas in very intelligible language, yet ſurviving only about 21 hours, I ſend you for inſertion in your miſcellany,

---

* The Diſtemper not having yet obtained any noſological name, perhaps that of Catarrhus Trachealis might not be deemed an improper one.

