# Addition to Mr. Forbes's Paper on the Distemper of Dogs

**Published:** 1801-11

**Authors:** 


*Addition to Mr.* Forbes's *Paper on the Distemper of Dogs.* Page 315.

The tranfcriber of Mr. Forbes's notes having omitted a part of the diffection, Mr. F. will be obliged to Dr. Bradley to add, after the words "the membrane on the right fide did not deviate from its natural ftate;" the left lobes of the lungs were turgid, and had tubercles, while thofe of the right feemed to have fuffered no change.

